# The Developmental Stage Symbionts of the Pea Aphid-Feeding *Chrysoperla sinica* (Tjeder)

**DOI:** 10.3389/fmicb.2019.02454

**Published:** 2019-11-01

**Authors:** Chenchen Zhao, Hui Zhao, Shuai Zhang, Junyu Luo, Xiangzhen Zhu, Li Wang, Peng Zhao, Hongxia Hua, Jinjie Cui

**Affiliations:** ^1^State Key Laboratory of Cotton Biology, Institute of Cotton Research, Chinese Academy of Agricultural Sciences, Anyang, China; ^2^Hubei Insect Resources Utilization and Sustainable Pest Management Key Laboratory, College of Plant Science and Technology, Huazhong Agricultural University, Wuhan, China; ^3^Zhengzhou Research Base, State Key Laboratory of Cotton Biology, Zhengzhou University, Zhengzhou, China

**Keywords:** *Chrysoperla sinica*, development stage, microbiotas, high-throughput sequencing, bacterial diversity

## Abstract

*Chrysoperla sinica* (Tjeder) is widely recognized as an important holometabolous natural enemy of various insect pests in different cropping systems and as a non-target surrogate in environmental risk assessment of Bt rice (i.e., genetically modified rice to express a toxin gene from *Bacillus thuringiensis*). Like other complex organisms, abundant microbes live inside *C. sinica*; however, to date, microbiome composition and diversity of the whole life cycle in *C. sinica* has not yet been well characterized. In the current study, we analyze the composition and biodiversity of microbiota across the whole life cycle of *C. sinica* by using high-throughput Illumina sequencing of the 16S ribosomal RNA gene. Collectively, Proteobacteria and Firmicutes dominated the microenvironment at all stages, but their relative abundances fluctuated by host developmental stage. Interestingly, eggs, neonates, and adults shared similar microbes, including an abundance of *Rickettsia* and *Wolbachia*. After larva feeding, *Staphylococcus*, Enterobacteriaceae, and *Serratia* were enriched in larvae and pupa, suggesting that food may serve as a major factor contributing to altered microbial community divergence at different developmental stages. Our findings demonstrated that *C. sinica* harbor a variety of bacteria, and that dynamic changes in community composition and relative abundances of members of its microbiome occur during different life cycle stages. Evaluating the role of these bacterial symbionts in this natural enemy may assist in developing environmental risk assessments and novel biological control strategies.

## Introduction

Mutualistic relationships with microbial symbionts have been observed in many insects and are often critical for their survival and development (Kikuchi et al., 2007; Moran et al., 2008; Santos-Garcia et al., 2017). Endosymbionts may profoundly influence insect ecology and evolution and drive the coevolution between plants and herbivores (Duron et al., 2008; Kikuchi et al., 2012; Pietri and Liang, 2018). Furthermore, bacterial symbionts may provide supplemental nutrients, protection from natural enemies, and defense against pathogens and facilitate tolerance of abiotic stress (Rolff and Siva-Jothy, 2003; Scarborough et al., 2005; Douglas, 2009; Gerardo and Parker, 2014; Heyworth and Ferrari, 2015). In addition, symbionts have been shown to play key roles in insect development but the underlying mechanisms are not well understood (Hammer et al., 2017). For example, genetic studies have provided evidence of asymmetric gene transfer from bacteria and fungi to *Bombyx mori* (silkworm), leading to its increased survival, and reproduction (Sun et al., 2013). Understanding the composition and function of bacterial symbionts and consequential effects on their hosts remain a significant challenge.

The green lacewing (*Chrysoperla sinica* Tjeder, Chrysopidae: Neuroptera) is widely recognized as a natural enemy of insect pests that are important to various cropping systems (e.g., maize–wheat–maize, cotton–wheat, and wheat–rice). Adult green lacewings are frequently employed for augmentation of biological control (McEwen, 2010; Liu et al., 2011b; Ragsdale et al., 2011). Honey-dew and floral nectar are the preferred food for adult lacewings, while nymphs feed on aphids, planthoppers, coccids, mites, whiteflies, eggs, and early-instar larvae of lepidopterans, as well as many other soft-bodied arthropods (Winterton and Freitas, 2010; Xu et al., 2010). For example, one *C. sinica* larva can prey on as many as 164 *Frankliniella occidentalis* nymphs or 238 *Bemisia tabaci* nymphs per day (Lin et al., 2002; Zhang et al., 2007). Moreover, *C. sinica* has been selected as a surrogate species in the environmental risk assessment of Bt rice because *in vitro* rearing and manipulation of *C. sinica* is not difficult (Wang et al., 2012; Li et al., 2013, 2014; Romeis et al., 2013). Very few studies have explored the gut bacteria of adult *C. sinica* (Liu et al., 2012). The symbiotic bacteria of *C. sinica* have rarely been utilized experimentally and their effects on developmental stages are not well understood (Liu et al., 2012; Zhao et al., 2017).

*Chrysoperla sinica* undergoes differentiated complete metamorphosis where larvae and adults have distinct form and function. To investigate how the microbial community associated with *C. sinica* varies with life stages, we used high-throughput sequencing of the 16S ribosomal RNA (16S rRNA) gene to conduct a systematic evaluation of the microbial communities associated with *C. sinica*. The results yielded insights into the influence of microbial symbionts on this important natural enemy, and helped to clearly define the symbiotic changes during the development of this insect.

## Materials and Methods

### Insect Rearing and Maintenance

*Chrysoperla sinica* adults were originally collected from cotton plants cultivated at the Institute of Cotton Research, Chinese Academy of Agricultural Sciences (36°5′34.8″N, 114°31′47.19″E). They were then maintained in an environmental chamber and supplied with 10% honey solution, whereas larvae were maintained on a diet of the pea aphid *Acyrthosiphon pisum*. Rearing conditions were 25 ± 1°C, 65 ± 5% relative humidity, and a 14:10 h light:dark cycle (the suitable environment for feeding) (Li Z. et al., 2015). Generation 6 (G6) *C. sinica* eggs were used as the starting point for all experiments.

### Sampling, DNA Extraction, and 16S rRNA Amplification Sequencing

Eggs, neonates, 1st instar larvae, 2nd instar larvae, 3rd instar larvae, pupae, and adults of *C. sinica* were randomly drawn from colonies. These seven treatments (stages) were applied with five replicates (each replicate had either 20 insects or 45 eggs). DNA was extracted from each group (whole body—each sample was washed for 5 min in 70% ethanol and rinsed three times with sterile water) using the TIAGEN DNeasy kit (TIANGEN Biotech, Beijing) following the manufacturer’s protocol. We supplemented tissue and cell lysis solutions with lysozyme of 50 mg/ml for efficient extraction. Insect homogenates were incubated at 37°C for 30 min. The quantity and quality of the DNA were measured with a NanoDrop 2000C spectrophotometer (Thermo Fisher Scientific, United States). The V3–V4 hypervariable region of the 16S rRNA gene of bacteria was amplified with the following PCR-cycling conditions: 3 min of denaturation at 95°C, 35 cycles of melting at 95°C for 30 s, annealing at 50°C for 30 s, extension at 72°C for 45 s, and a final extension at 72°C for 10 min, and with the universal primers (338F: 5′-ACTCCTACGGGAGGCAGCAG-3′, 806R: 5′-GGACTACHVGGGTWTCTAAT-3′) that were designed for detecting a broad range of microorganisms (Xu et al., 2016). PCR (GeneAmp 9700, ABI, United States) reactions were performed in triplicate 20-μl mixture containing 4 μl of 5 × FastPfu Buffer, 2 μl of 2.5 mM dNTPs, 0.8 μl of each primer (5 μM), 0.4 μl of FastPfu Polymerase, and 10 ng of template DNA. PCR reactions were subjected to 2% agarose gel electrophoresis. Amplicons of the expected size were excised, purified with the AxyPrep DNA Gel Extraction Kit (Axygen Biosciences, Union City, CA, United States), and quantified with QuantiFluorTM-ST (Promega, United States) according to the manufacturer’s protocol. Then, purified amplicons were pooled at equimolar concentrations (>2 nM) (TruSeq^TM^ DNA Sample Prep Kit was used for library construction) and paired-end sequenced (2 × 300 bp, with an insert size of 468 bp) on an Illumina MiSeq platform (Illumina, San Diego, CA, United States) at Shanghai Majorbio Bio-pharm Technology Co., Ltd. The sequences obtained in this study were deposited in the GenBank short-read archive (SRA), accession number PRJNA531272^1^.

### High-Throughput Analysis

Raw fastq files were quality-filtered via Trimmomatic and merged by FLASH^2^ with the following criteria: (i) The reads were truncated at any site that received an average quality score <20 over 50 bp (determined by sliding window). (ii) Sequences whose overlaps were longer than 10 bp were merged (allowing two nucleotide mismatching). (iii) Sequences of each sample were separated according to barcodes (exactly matching) and primers (allowing two nucleotide mismatching), and reads containing ambiguous bases were removed. High-quality reads were analyzed using both QIIME (version 1.6.0) (Caporaso et al., 2010) and mothur (version 1.30.1) (Schloss et al., 2009). Reads were assigned to their designated sample and then length-based filtering (<200 bp was excluded from the analysis) and read-quality filtering were performed (using the denoise_wrapper.py script in QIIME) (Chen et al., 2016). Operational taxonomic units (OTUs) were clustered at 97% similarity (Edgar, 2013). The taxonomy of each 16S rRNA gene sequence was analyzed by Performances of Ribosomal Database Project (RDP) Classifier algorithm (vision 2.2^3^) against the SILVA 16S rRNA database (Release128^4^) (Quast et al., 2013) using a confidence threshold of 70%. Based on the resulting OTU table, we tested for the presence of enterotypes (Li J. et al., 2015) (which were introduced to reflect proportional categories or patterns within the microbiota in different individuals). Samples were clustered using the Jensen–Shannon distance and partitioning around medoid (PAM) clustering. The optimal number of clusters was estimated using the Calinski-Harabasz (CH) index (Arumugam et al., 2011; Koren et al., 2013), and principal coordinates analysis (PCoA, *K* ≥ 2) was carried out for visualization (Wu et al., 2011).

To avoid heterogeneity among samples due to repeated numbers of samples, and for richness and diversity analyses, rarefied OTUs were sampled. Chao1 and Ace estimators and Shannon and Simpson indices were also calculated for diversity. For normally distributed data, one-way ANOVAs followed by LSD test were performed to compare between treatments. Non-normally distributed samples were subjected to the Kruskal–Wallis test to compare between treatments. Normality was tested using the Shapiro-Wilk test. *P* values ≤ 0.05 were considered statistically significant. These analyses were performed with the SAS 9.4 software package. For group comparisons, the abovementioned rarefied OTU tables were used to construct Bray–Curtis matrices. Principal coordinate analysis was used for visualization using community membership and relatedness of community members (Lozupone et al., 2011). UniFrac was used for microbial community comparison (Hamady et al., 2010; Lozupone et al., 2011). Using *ggplot2* package in *R*, taxonomic heat maps were constructed using a complete linkage clustering method and Bray–Curtis distance metric (Ginestet, 2011). Detailed methods are in the Supplementary  Materials.

## Results

### Overview of *C. sinica* Microbiotas

**FIGURE 1:**
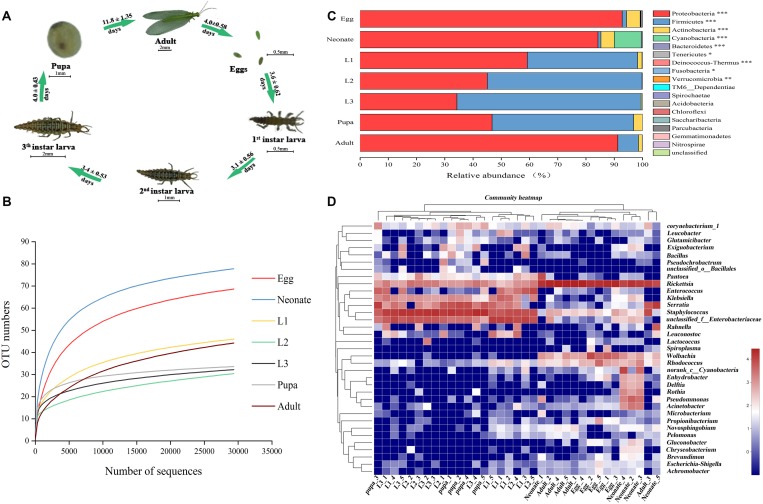
Bacterial community dynamics among different developmental stages in *Chrysoperla sinica.* **(A)** Overview of development stages of the *C. sinica*. **(B)** Rarefaction curves constructed from randomly subsampled data sets with the same number of 16S sequences. The near-saturated rarefaction curve indicates that the vastness of microbial diversity was captured from each sample. **(C)** Relative abundance of bacteria communities at the phylum level in different groups (Non-parametric Kruskal–Wallis test ^∗^0.01 < *P* ≤ 0.05, ^∗∗^0.001 < *P* ≤ 0.01, and ^∗∗∗^*P* ≤ 0.001). **(D)** Heat map of major taxa over the life cycle at the genus level. Cluster analysis using the Bray–Curtis distance and the complete-linkage method. Each column represents a single replicate of each of the seven treatments. Columns were clustered according to the similarity of bacterial abundance profiles. Each row represents an OTU assigned to the genus level. Color gradient represents the proportion of species. Plotting scale, from red to blue, indicates the decrease in richness of bacterial communities. Five replicates are labeled 1–5.

Like all holometabolous insects, *C. sinica* undergoes complete metamorphosis (Figure 1A). To assess associated microbial communities, DNA from whole insects at different developmental stages (egg; neonate; 1st, 2nd, and 3rd instars; pupa; and adult) was extracted and Illumina sequence of bacterial 16S rRNA was analyzed. Community diversity and composition were analyzed using deep sequencing. We generated 1,729,032 raw reads, 1,340,914 high-quality reads, 590,093,503 base sequence, and an average of 440.07 length (Supplementary Table S1). The sequences were clustered into 262 OTUs that shared ≥97% sequence identity (Supplementary Table S1). A total of 26 OTUs were shared among all seven groups (samples shared 3 OTUs) (Supplementary Table S2).

### Taxonomic Classification of Microbiota Among Different Development Stages in *C. sinica*

To assess whether sampling was adequate, we used a rarefaction curve, which indicated that we captured the majority of microbial diversity in all samples (Figure 1B). Taxonomic analysis of all samples showed that Proteobacteria was the most prevalent phylum. In all samples, the top three phyla with highest relative abundances were Proteobacteria, Firmicutes, and Actinobacteria (Figure 1C and Supplementary Table S3). Regardless of age or host species, bacterial ratios varied considerably among the groups. The prevalence of Proteobacteria was significantly higher in eggs (92.69%), neonates (82.40%), and adults (92.12%), while it decreased sharply in the 1st instar larvae (59.26%). The most prevalent phylum in the larvae was Firmicutes, which reached a maximum abundance in the 3rd instar larvae (64.91%). The prevalence of Firmicutes decreased in pupae corresponding with an increase in Proteobacteria (45.63%). After eclosion, relative abundance of Firmicutes decreased (6.50%) in adults while the prevalence of proteobacteria increased. Proteobacteria, Firmicutes, and Actinobacteria showed significantly different relative abundances across a complete life cycle (*P* < 0.001, Supplementary Table S3).

A heat map was generated to visualize the distribution of multiple OTUs in different groups (Figure 1D). The assigned heat map corresponding to the genus level (35 most abundant) presented a comprehensive overview of the bacterial community composition. Some bacterial taxa were consistent colonizers of *C. sinica*, including *Rickettsia* and *Staphylococcus*. Eggs shared a similar composition of bacterial types compared with neonates and adults, whereas the abundance in larvae appeared to be similar to pupa. *Rickettsia* was the most abundant bacterial genus in eggs (76.16%), neonates (46.97%), and adults (85.57%). The bacterial community in neonate adults had a greater diversity relative to that of the 1st instar, and this may be the result of feeding. Interestingly, most bacteria were also found to be associated with egg masses, indicating that certain bacteria might be transmitted to the filial generation. In contrast, *Staphylococcus*, *Enterococcus*, and *Klebsiella* had the highest richness of all bacterial taxa in larvae and pupae. *Serratia* also comprised a major microbial component of pupae (29.73%), but comprised a small portion of other developmental stages (4.04–9.53%), and a very low abundance in eggs (0.05%). After adult eclosion, a significant rise in the abundance of *Rickettsia* and a decrease of *Staphylococcus* was observed. Eggs harbored a great abundance of *Wolbachia* (15.38%), with lower frequencies found in neonates (2.67%) and adults (1.80%). *Pseudomonas* had a high abundance in neonates and was rarely detected in other developmental stages. Furthermore, some groups (e.g., *Leuconstoc*) were only present in different stages of larval development. Therefore, it can be concluded that bacterial community composition largely shifted between 1st instars feeding and adult eclosion.

### Comparative Assessment of the Microbiota Among Different Developmental Stages

We analyzed α-diversity using several methods, i.e., OTU species counts, ACE index for microbial richness, Chao1 index, the Shannon index, Simpson index, and the Good’s coverage for community coverage (Supplementary Table S1). Good’s coverage is an estimation of the percentage of the total species represented in a sample, and we found an average coverage of 99.98%, which suggested that most of the bacterial phylotypes in the insects were represented in this study. However, the *C. sinica*-associated bacterial community (designated as microbiota) was observed to be variable across all life stages of the host.

**FIGURE 2:**
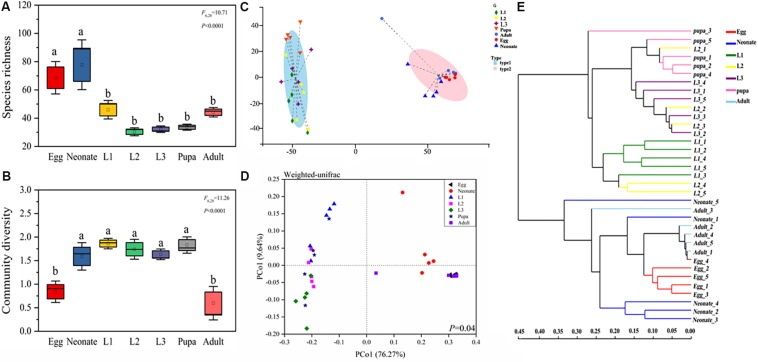
Bacterial community dynamics among different developmental stages in *C. sinica*. Boxplot of species richness (number of OTUs) **(A)** and community diversity measured by the Shannon index **(B)**. Different lowercase labels above each group indicates significant differences (one-way ANOVA, LSD *post hoc* test, *P* < 0.05) of group mean value. **(C)** Enterotype analysis of OTU level according to β-diversity metrics of UniFrac. **(D)** Principal coordinates analysis (PCoA) plot visualizing the data based on β-diversity metrics of UniFrac (NMDS analysis *P* ≤ 0.05). **(E)** Unweighted pair-group method with arithmetic means (UPGMA) analysis of microbial community structure based on 16S rRNA gene amplicon sequencing data.

Diversity and richness of bacterial species were found to be significantly different among developmental stages of *C. sinica*. Egg and neonate had more bacterial species, with 72 unique OTUs and an average of 80 OTUs per sample, while significantly lower species richness was observed in larvae and pupa. Taxon number was lowest in the 2nd instar larvae (32 unique OTUs per sample on average). After metamorphosis, bacterial species richness increased slightly in adults (and 1st instar). Eggs and adults harbored lower diversity (Figures 2A,B), while other developmental stages had a higher diversity of bacterial species. Similarly, Simpson diversity, Chao1, and Ace richness estimators, all exhibited similar patterns (Supplementary Figure S1). Neonates harbored higher species richness and diversity than the other stages: neonates > eggs > 1st instar larvae > adults > pupa > 3rd instar larvae > 2nd instar larvae. The community diversity exhibited the following pattern: 1st instar larvae > pupa > 2nd instar larvae > 3rd instar larvae > neonates > eggs > adults.

The enterotype analysis suggested that the bacterial colonies in *C. sinica* were divided into two types (Figure 2C). Larvae and pupa were grouped with one enterotype, whereas eggs, neonates, and adults were grouped with a different enterotype. Similar results were also found in our principal coordinate analysis (Figure 2D), which showed relatively tight clustering of individuals within the same developmental stage, although differences between growth stages were also observed. The results of the unweighted pair-group method using arithmetic averages (UPGMA) analysis indicated two clusters. Cluster I contained four developmental stages (1st instar larva, 2nd instar larva, 3rd instar larva, and pupa), while Cluster II contained three stages (egg, neonate, and adult), which were associated with diet as opposed to developmental stage (larvae fed by pea aphid; adults fed on honey solution; other stages fed on nothing). This suggests that the main factor determining microbial community divergence in different stages is aphids. The PCoA, enterotype, and cluster analysis results indicated that egg and neonate were more similar to adult, while larva were more similar to pupa. These results are consistent with the abovementioned alpha analysis. The 2nd instar acted as a transitional period connecting 1st instar and 3rd instar. Changes in the community composition were correlated with feeding habit as opposed to molting, suggesting that feeding habit drives the microbial community composition.

Dendrograms were generated to illustrate the composition and relatedness of bacterial communities from *C. sinica* based on OTU information (Figure 2E). As shown by the cluster analysis, there were two branches, the first branch consisted of eggs, neonates, and adults, while the second branch consisted of larvae and pupae. In general, the distances of samples in the same branch were within 0.35 of each other (a value of 0 means they have the same composition and a value of 1 means they do not share any species), which indicates that larvae and pupae, eggs, neonates, and adults shared a high similarity in their bacterial communities.

## Discussion

Although microbial diversity in insects has been widely studied, most studies have been taxon specific and focused on the insect gut (Roh et al., 2008; Hulcr et al., 2012; Salem et al., 2013). In the current study, we present a comprehensive examination of the diverse populations of bacteria found in the lacewing *C. sinica* during its life cycle by using next-generation sequencing. We found that Proteobacteria and Firmicutes were dominant across the entire life cycle, which was similar to observations in other insects (Colman et al., 2012; Yun et al., 2014), such as *B. mori* (Chen et al., 2018), *B. tabaci* (Gottlieb et al., 2006), *Drosophila melanogaster* (Chandler et al., 2011), and *Apis mellifera* (Engel et al., 2012). Similar results were also found in other species, including *Mytilus coruscus* (Li et al., 2018), *Lytechinus variegatus* (Hakim et al., 2015), and humans (Schnorr et al., 2014). Despite significant diversity in the initial developmental stages (egg and neonate mass), significant reductions in bacterial diversity was observed during later developmental stages of *C. sinica* from eggs to adults (Figure 1C). Overall, the microbiota of *C. sinica* exhibited low diversity compared to the microbiota of other insects, such as *Odontotaenius disjunctus* (Ceja-Navarro et al., 2014) and *Lymantria dispar* (Mason and Raffa, 2014). By consuming large quantities of prey, lacewing larvae may ingest large amounts of potentially harmful microbes. It would thus make sense for such a voracious host to efficiently control its microbiota and quickly clear invading microbes (Chen et al., 2016). Such a phenomenon could explain the relatively simple microbiota observed in *C. sinica*.

In different developmental stages, the relative abundance and diversity indices varied across stages. In the early stages of development, species richness of bacteria was greater than in the later stages. In contrast, community diversity was greater in the later than in the earlier stages. Because *C. sinica* is holometabolous, the transition from egg to adult comprises complex changes in structural variation (Figure 1A). The difference in the structure and diversity of bacteria may be due to different physiological states and metabolism of *C. sinica* at different ages. Microbial symbionts that affect developmental, physiological, and ecological interactions are present in many animals (Hammer et al., 2017).

A shift in the dominant (most abundant) taxa across developmental stages was identified. From *Rickettsia* and *Wolbachia* (Alpha-Proteobacteria) in eggs, neonates, and adults to *Staphylococcus* (Firmicutes), and Enterobacteriaceae and *Serratia* (Gamma-Proteobacteria) in larvae and pupae. All these dominant taxa have been frequently detected in other insects (Hosokawa et al., 2010; Brumin et al., 2011; Haloi et al., 2016). Thermotolerance had been reported to be associated with *Rickettsia*, which has vertical transmission in invertebrates (Brumin et al., 2011). *Rickettsia* can also manipulate host reproduction in *Adalia decempunctata* (von der Schulenburg et al., 2001), *Brachys tessellates* (Lawson et al., 2001), *Coccotrypes dactyliperda* (Zchori-Fein et al., 2006), and *Neochrysocharis formosa* (Hagimori et al., 2006). *Wolbachia* are common transovarially transmitted symbionts of arthropods and nematodes (Baldo et al., 2006). In arthropods, *Wolbachia* have been shown to influence numerous aspects of host biology, including reproductive manipulation (e.g., male killing, feminization, sperm–egg incompatibility and parthenogenesis; Dobson et al., 2002; Bourtzis, 2008; Werren et al., 2008). *Wolbachia* can also provide benefits (Weeks et al., 2007) and can develop mutualistic relationships with their hosts (Hosokawa et al., 2010). Relative abundance of *Wolbachia* in eggs adults and neonates was much higher than other stages in current study. Similarly, *Wolbachia* abundance in adult *Aedes albopictus* is significantly higher than in larvae or pupae (Wang et al., 2018). Moreover, *Wolbachia* was shown to be the most dominant genus in pupa and adult stages of *Hoplothrips carpathicus*, while in larvae, it occurred at a lower frequency (Kaczmarczyk et al., 2018). The mechanism of observed disproportion is unclear. Food, development stage larvae and pupae immunity, incomplete maternal transmission, or selection pressure of *Wolbachia* could account for such a differential rate of infection on different stages in *C. sinica*. In the bed bug, *Cimex lectularius*, *Wolbachia* densities vary with life stage (high in adults; Fisher et al., 2018). Similarly, *Wolbachia* densities are altered by diet in *Drosophila* (Christensen et al., 2019). Infection by *Wolbachia* may provide a selective advantage for the female flies and little or no advantage for the males (*Wolbachia* can infect all females, but not all males in *Orseolia oryzae*) (Behura et al., 2001). Another study found that *Wolbachia* infection alters the relative abundance of resident bacteria in adult *Aedes aegypti*, but not larvae (Audsley et al., 2018), which could explain why *Wolbachia*-infected treatments share lower community diversity (Figure 1D). Pea aphid has been documented to perform as both host and vector for the phytopathogenic bacterium *Pseudomonas* (Stavrinides et al., 2009). In our study, *Pseudomonas* had high abundance in neonates, but was rarely detected in other developmental stages, suggesting that the neonate is unable to degrade prey-associated bacteria immediately.

The bacterial composition of adult *C. sinica* in our study differs from that reported by Liu et al. (2012) for field-collected adult *C. sinica* based on culture-dependent methods. Ten bacterial taxa, including *Morganella*, *Bacillus pumilus*, and *Terrabacter*, were isolated by Liu et al. (2012), few of which overlapped with the bacterial taxa detected in the present study. However, many of the microbial associates are not readily culturable. It is also well known that bacterial diversities vary from field-collected to lab-reared (Rani et al., 2009) as well as across different geographical regions (Zouache et al., 2011). In previous studies, the bacterial composition and diversity were influenced by the *in vitro* environment (Zouache et al., 2011), as well as medium selection and an artificial diet (Priya et al., 2012). In addition, microbial diversity could be affected by different physiological and pathological states of the host (Backhed et al., 2004; Ley et al., 2006). *C. sinica* is also affected by the ecological niche of its host, as well as the environment and its own physiological status (Xu et al., 2002, 2010; Khuhro et al., 2014). Age, mating status, temperature, and humidity affect flight performance and other behaviors (e.g., adult diapause, survival rate, and fecundity) of *C. sinica* (Xu et al., 2004, 2010; Liu et al., 2011a, b; Chen et al., 2017). Previous observations suggest that prey could alter pre-imaginal development, survival, adult longevity, and fecundity of *C. sinica* (Khuhro et al., 2012). Moreover, microbial diversity varies greatly among insects that have fed on different food sources, which has been shown for *Helicoverpa armigera* (Priya et al., 2012), *Lymantria dispar* (Broderick et al., 2004), and *Anopheles stephensi* (Rani et al., 2009). In particular, we were interested in whether or not food exerted an effect on the microbial diversity in *C. sinica*.

In this study, principal coordinate analysis revealed significant changes in the bacterial community compositions under different developmental stages, demonstrating that the variation between aphid feeding stage (initial stage of larvae feeding) and non-feeding stage (after adult eclosion to neonate) was greater than within the same stage. The 2nd instar stage acted as a transitional period that connected the 1st instar and 3rd instar. Although gut morphology was altered sharply by metamorphosis, during different stages of the life cycle (Moll et al., 2001), feeding, and food source may have caused the variation in host-specific microbiota in *C. sinica*.

The bacterial community exhibited by *C. sinica* probably results from flexible and facultative associations with free-living and horizontally transmitted bacteria, akin to the phenomenon reported in *Drosophila* (Chandler et al., 2011). In our study, we used the pea aphid, which is a highly palatable host that fosters lacewing ingestion and development. Previous studies have shown a community composition shift on less preferred species (Xiang et al., 2010).

Transgenic Bt cotton had little effect on the biological characteristics of *C. sinica* that fed on *A. gossypii* (Gao et al., 2013). In addition, the insecticidal protein Cry1Ab and Cry2Aa in rice pollen had no significant adverse effects on adult *C. sinica* (Bai et al., 2005; Wang et al., 2012). Yet, feeding on *Spodoptera exigua* that were reared on Bt cotton exerted some influence on the life history traits of *C. sinica* (Shi et al., 2011). Effects of Bt toxin on bacterial diversity of *A. mellifera* has been a theoretical concern for biosafety assessments of transgenic maize and cotton (Dirk et al., 2010; Weiyu et al., 2010; Geng et al., 2013; Jia et al., 2016). Similar investigations have been undertaken in *Nilaparvata lugens* (Gao et al., 2008), *Holotrichia oblita* (Wang et al., 2017), and *Chilo suppressalis* (Li et al., 2016). Despite being important as a surrogate species in environmental risk assessment, the bacterial diversity of *C. sinica* had not been well characterized. In the current study, we have provided a better understanding of the symbiont diversity and community composition patterns associated with different life cycle stage of *C. sinica*. Such knowledge may help advance the manipulation and exploitation of the microbiota, which could result in important practical applications for the development of strategies for the management of insect-related problems (Crotti et al., 2012). For instance, the effects of Bt protein on the bacterial communities of the coccinelid beetle *Propylea japonica* have been estimated, providing a new aspect of non-target risk assessment of genetically modified crops (Zhang et al., 2019). The present study will help lay the foundation for management of *C. sinica*-related problems in the future (e.g., the effects of genetically modified organisms on *C. sinica* bacterial communities).

## Data Availability Statement

Publicly available datasets were analyzed in this study. This data can be found here: https://www.ncbi.nlm.nih.gov/bioproject/?term=PRJNA531272.

## Author Contributions

JC and SZ conceived and designed the work, and edited the manuscript. CZ and HZ performed the experiments and wrote the manuscript. JL, XZ, and LW helped with the theoretical analysis. PZ and HH helped to revise the manuscript.

## Conflict of Interest

The authors declare that the research was conducted in the absence of any commercial or financial relationships that could be construed as a potential conflict of interest.

## Footnotes

1 https://www.ncbi.nlm.nih.gov/sra/?term=PRJNA531272

2 http://ccb.jhu.edu/software/FLASH/

3 http://sourceforge.net/projects/rdp-classifier/

4 http://www.arb-silva.de

## Supplementary Material

FIGURE S1 Bacterial community dynamics among *C. sinica* in different developmental stages. A Boxplot of species richness (Chao1 and Ace richness estimators) and community diversity (Simpson diversity index). Different lowercase above each host species indicate significant differences (one-way ANOVA, LSD *post-hoc* test, *P* < 0.05) in the mean values.

TABLE S1 Summary of high-throughput sequencing read analysis, microbial community diversity richness (OTUs, 97%), sample coverage (Good’s coverage), diversity index (Shannon, ACE, Shannon, Simpson), and estimated OTU richness (Chao1) for community diversity analyses of 35 samples from *C. sinica* of different development stages.

TABLE S2 Details of bacterial OTUs shared in *C. sinica* among different developmental stages.

TABLE S3 Relative abundance of bacteria communities at the phylum level in different group.

TABLE S4 Supplementary methods, bioinformatics analysis.
