# Supplementary material for: The Developmental Stage Symbionts of the Pea Aphid-Feeding Chrysoperla sinica (Tjeder)
Source: Front Microbiol. 2019 Nov 1;10:2454. doi: 10.3389/fmicb.2019.02454 (PMC6839393; doi:10.3389/fmicb.2019.02454)
Supplement: TABLE S1 — Summary of high-throughput sequencing read analysis, microbial community diversity richness (OTUs, 97%), sample coverage (Good’s coverage), diversity index (Shannon, ACE, Shannon, Simpson), and estimated OTU richness (Chao1) for community diversity analyses of 35 samples from C. sinica of different development stages. [file Table_1.DOCX]

Supplement Table 1 Summary of high-throughput sequencing read analysis, microbial community diversity richness (OTUs, 97%), sample coverage (Good's coverage), diversity index (Shannon, ACE, Shannon, Simpson), and estimated OTU richness (Chao1) for community diversity analyses of 35 samples from *C. sinica* of different development stages.

|  |  | | | | **Diversity index** | | |  |  | | |
| --- | --- | --- | --- | --- | --- | --- | --- | --- | --- | --- | --- |
|  | **Sequences**  **number** | **Mean length** | **OTUs** | **Phylum** | **Genus** | **Shannon** | **Chao1** | **ACE** | **Simpson** | **PD** | **Coverage** |
| **Egg_1** | 38188 | 426.24 | 86 | 10 | 70 | 0.5988 | 89.9 | 91.54 | 0.5988 | 11.15 | 0.9996 |
| **Egg_2** | 38342 | 425.82 | 61 | 11 | 51 | 0.5001 | 68.1 | 70.30 | 0.5001 | 7.28 | 0.9996 |
| **Egg_3** | 30413 | 426.29 | 60 | 8 | 46 | 0.6581 | 81.0 | 76.65 | 0.6581 | 6.56 | 0.9995 |
| **Egg_4** | 43739 | 425.39 | 51 | 6 | 40 | 0.8944 | 53.6 | 59.80 | 0.8944 | 5.85 | 0.9997 |
| **Egg_5** | 41817 | 426.64 | 86 | 12 | 73 | 0.4803 | 86.4 | 86.76 | 0.4803 | 10.90 | 0.9999 |
| **Neonate_1** | 39917 | 433.00 | 52 | 6 | 47 | 0.4687 | 65.0 | 74.29 | 0.4687 | 6.68 | 0.9995 |
| **Neonate_2** | 32882 | 433.49 | 91 | 7 | 62 | 0.3865 | 100.2 | 96.62 | 0.3865 | 7.83 | 0.9996 |
| **Neonate_3** | 52076 | 432.36 | 104 | 6 | 73 | 0.2486 | 111.5 | 108.92 | 0.2486 | 10.31 | 0.9997 |
| **Neonate_4** | 41257 | 430.50 | 90 | 8 | 61 | 0.2572 | 94.0 | 94.96 | 0.2572 | 8.28 | 0.9997 |
| **Neonate_5** | 30967 | 441.66 | 51 | 7 | 43 | 0.3480 | 57.9 | 60.43 | 0.3480 | 6.06 | 0.9996 |
| **L1_1** | 37792 | 449.10 | 43 | 4 | 33 | 0.2467 | 54.0 | 58.45 | 0.2467 | 4.04 | 0.9996 |
| **L1_2** | 52531 | 449.10 | 55 | 7 | 47 | 0.1782 | 66.0 | 64.29 | 0.1782 | 7.17 | 0.9996 |
| **L1_3** | 36567 | 448.63 | 50 | 5 | 37 | 0.2649 | 51.9 | 53.30 | 0.2649 | 5.20 | 0.9998 |
| **L1_4** | 43659 | 449.31 | 35 | 4 | 27 | 0.1826 | 44.2 | 63.87 | 0.1826 | 3.70 | 0.9996 |
| **L1_5** | 33927 | 449.03 | 52 | 6 | 42 | 0.1722 | 61.1 | 66.21 | 0.1722 | 6.16 | 0.9995 |
| **L2_1** | 38732 | 447.99 | 25 | 4 | 19 | 0.2459 | 28.0 | 28.90 | 0.2459 | 2.97 | 0.9999 |
| **L2_2** | 32919 | 447.73 | 33 | 5 | 25 | 0.3321 | 44.0 | 72.80 | 0.3321 | 4.18 | 0.9996 |
| **L2_3** | 31792 | 447.15 | 26 | 5 | 20 | 0.3613 | 31.6 | 33.06 | 0.3613 | 3.60 | 0.9997 |
| **L2_4** | 33348 | 449.06 | 34 | 5 | 25 | 0.1548 | 41.0 | 49.23 | 0.1548 | 3.83 | 0.9997 |
| **L2_5** | 44118 | 448.00 | 33 | 6 | 23 | 0.2128 | 38.6 | 39.65 | 0.2128 | 4.26 | 0.9997 |
| **L3_1** | 44472 | 448.01 | 35 | 4 | 24 | 0.2353 | 44.0 | 63.46 | 0.2353 | 3.47 | 0.9997 |
| **L3_2** | 30694 | 447.97 | 31 | 4 | 23 | 0.3481 | 34.8 | 38.29 | 0.3481 | 3.47 | 0.9998 |
| **L3_3** | 42151 | 447.99 | 33 | 3 | 24 | 0.3662 | 66.0 | 61.96 | 0.3662 | 2.91 | 0.9996 |
| **L3_4** | 34152 | 447.35 | 36 | 4 | 28 | 0.3387 | 38.0 | 40.03 | 0.3387 | 3.68 | 0.9998 |
| **L3_5** | 38365 | 449.43 | 33 | 4 | 24 | 0.3126 | 38.0 | 36.69 | 0.3126 | 3.16 | 0.9998 |
| **Pupa_1** | 39837 | 448.92 | 33 | 4 | 26 | 0.2939 | 35.5 | 43.63 | 0.2939 | 3.19 | 0.9998 |
| **Pupa_2** | 30783 | 448.47 | 30 | 3 | 23 | 0.2649 | 30.5 | 30.89 | 0.2649 | 2.71 | 0.9999 |
| **Pupa_3** | 33700 | 448.64 | 38 | 3 | 25 | 0.1636 | 40.5 | 41.95 | 0.1636 | 2.88 | 0.9998 |
| **Pupa_4** | 33994 | 448.39 | 31 | 3 | 24 | 0.2610 | 31.5 | 31.83 | 0.2610 | 2.53 | 0.9999 |
| **Pupa_5** | 42618 | 447.86 | 36 | 3 | 24 | 0.3596 | 46.5 | 48.31 | 0.3596 | 2.79 | 0.9998 |
| **Adult_1** | 40050 | 425.33 | 45 | 7 | 35 | 0.9111 | 52.1 | 65.62 | 0.9111 | 5.57 | 0.9996 |
| **Adult_2** | 38430 | 426.72 | 44 | 6 | 36 | 0.8112 | 53.2 | 52.60 | 0.8112 | 5.06 | 0.9996 |
| **Adult_3** | 33556 | 437.94 | 36 | 6 | 30 | 0.2909 | 39.5 | 41.10 | 0.2909 | 4.64 | 0.9998 |
| **Adult_4** | 44473 | 425.78 | 54 | 7 | 46 | 0.9069 | 60.0 | 63.08 | 0.9069 | 6.87 | 0.9996 |
| **Adult_5** | 38656 | 425.32 | 37 | 7 | 31 | 0.8765 | 43.0 | 45.21 | 0.8765 | 4.82 | 0.9997 |
