# Supplementary material for: The Developmental Stage Symbionts of the Pea Aphid-Feeding Chrysoperla sinica (Tjeder)
Source: Front Microbiol. 2019 Nov 1;10:2454. doi: 10.3389/fmicb.2019.02454 (PMC6839393; doi:10.3389/fmicb.2019.02454)
Supplement: TABLE S2 — Details of bacterial OTUs shared in C. sinica among different developmental stages. [file Table_2.DOCX]

Supplementary Table 2 Details of bacterial OTUs shared in *C. sinica* among different developmental stages

| OTU ID | Phylum | Genus |
| --- | --- | --- |
| 41 | **Proteobacteria** | ***Rickettsia*** |
| 21 | **Firmicutes** | ***Staphylococcus*** |
| 31 | **Firmicutes** | ***Staphylococcus*** |
| 150 | Actinobacteria | *Rhodococcus* |
| 33 | Proteobacteria |  |
| 200 | Actinobacteria | *Corynebacterium* |
| 51 | Proteobacteria |  |
| 108 | Cyanobacteria | *norank_c__Cyanobacteria* |
| 27 | Firmicutes | *Enterococcus* |
| 85 | Proteobacteria | *Klebsiella* |
| 25 | Proteobacteria | *Enterobacter* |
| 34 | Proteobacteria | *Serratia* |
| 91 | Proteobacteria | *Acinetobacter* |
| 32 | Proteobacteria | *Serratia* |
| 69 | Proteobacteria | *Pantoea* |
| 39 | Proteobacteria | *Novosphingobium* |
| 260 | Actinobacteria | *Arthrobacter* |
| 15 | Firmicutes | *Bacillus* |
| 3 | Firmicutes | *Enterococcus* |
| 197 | Firmicutes | *Enterococcus* |
| 30 | Proteobacteria | *Rickettsia* |
| 14 | Firmicutes | *Leuconostoc* |
| 255 | Actinobacteria | *Leucobacter* |
| 66 | Actinobacteria | *Microbacterium* |
| 256 | Actinobacteria | *Corynebacterium* |
| 18 | Firmicutes | *Tetragenococcus* |
