# Supplementary material for: The Developmental Stage Symbionts of the Pea Aphid-Feeding Chrysoperla sinica (Tjeder)
Source: Front Microbiol. 2019 Nov 1;10:2454. doi: 10.3389/fmicb.2019.02454 (PMC6839393; doi:10.3389/fmicb.2019.02454)
Supplement: TABLE S3 — Relative abundance of bacteria communities at the phylum level in different group. [file Table_3.DOCX]

Supplementary Table 3 Relative abundance of bacteria communities at the phylum level in different group

|  | Egg | Neonate | L1 | L2 | L3 | Pupa | Adult |
| --- | --- | --- | --- | --- | --- | --- | --- |
| Proteobacteria | 0.92692 | 0.82399 | 0.59257 | 0.44952 | 0.34612 | 0.45628 | 0.9212 |
| Firmicutes | 0.01443 | 0.01151 | 0.39002 | 0.54888 | 0.64907 | 0.51403 | 0.06495 |
| Actinobacteria | 0.05073 | 0.05288 | 0.01573 | 0.00124 | 0.00475 | 0.02944 | 0.01309 |
| Cyanobacteria | 7.77E-04 | 0.10668 | 0.00136 | 2.37E-05 | 5.55E-06 | 2.44E-04 | 1.50E-04 |
| Bacteroidetes | 0.00178 | 0.00395 | 2.41E-04 | 3.20E-04 | 2.22E-05 | 0 | 2.59E-04 |
| Tenericutes | 0.00432 | 6.19E-05 | 0 | 0 | 0 | 0 | 1.66E-04 |
| Deinococcus-Thermus | 3.06E-04 | 8.41E-04 | 0 | 1.18E-05 | 0 | 0 | 1.35E-04 |
| Verrucomicrobia | 1.43E-04 | 0 | 5.03E-06 | 0 | 0 | 0 | 0 |
| TM6__Dependentiae | 1.48E-04 | 0 | 0 | 0 | 0 | 0 | 0 |
| Spirochaetae | 9.51E-05 | 0 | 0 | 0 | 0 | 0 | 1.04E-05 |
| others | 3.49E-04 | 8.77E-05 | 7.04E-05 | 0 | 3.33E-05 | 5.96E-06 | 2.59E-05 |
