# Supplementary material for: The Developmental Stage Symbionts of the Pea Aphid-Feeding Chrysoperla sinica (Tjeder)
Source: Front Microbiol. 2019 Nov 1;10:2454. doi: 10.3389/fmicb.2019.02454 (PMC6839393; doi:10.3389/fmicb.2019.02454)
Supplement: TABLE S4 — Supplementary methods, bioinformatics analysis. [file Table_4.DOCX]

**Bioinformatics analysis**

1. **Data Management**
   1. Raw data statistics

Image data output from sequencing machine is transformed by base calling into sequence data, which is called raw data (or raw reads) and stored in fastq format. Each sample used paired-end sequencing has two raw data files, named fq1 and fq2, respectively. Sequences obtained in this study were deposited in the GenBank short-read archive (SRA), accession number PRJNA531272 (<https://www.ncbi.nlm.nih.gov/sra/?term=PRJNA531272>).

- 1. Optimized data statistics

Illumina MiSeq sequencing yields pair-end sequence data. First, the pair-end reads are merged into a sequence according to their overlap relationship, and then the result of the merge is evaluated. Meanwhile, the quality filter of reads is conducted. Distinguish samples based on the barcodes and primer sequences at both ends of the sequence and obtain valid sequences. After correcting the direction of the sequence, optimized data is obtained. Raw fastq files were quality-filtered via Trimmomatic and merged by FLASH (<http://ccb.jhu.edu/software/FLASH/>). Data decontamination methods and parameters:

1. The reads were truncated at any site that received an average quality score <20 over a 50 bp (determined by sliding window).
2. Sequences whose overlaps were longer than 10 bp were merged (allowing 2 nucleotide mismatching).
3. Sequences of each sample were separated according to barcodes (exactly matching) and primers (allowing 2 nucleotide mismatching) and reads containing ambiguous bases were removed.
4. **Taxon Anno and Assess**
   1. OUT analysis

Operational Taxonomic Unit (OTU) is an operational definition used to classify groups of closely related individuals, and an OTU is simply the group of organisms currently being studied. Nowadays, however, the term "OTU" is generally used in a different context and refers to clusters of (uncultivated or unknown) organisms, grouped by DNA sequence similarity of a specific taxonomic marker gene. For several years, OTUs have been the most commonly used units of microbial diversity, especially when analyzing small subunit 16S or 18S rRNA marker gene sequence datasets. Sequences can be clustered according to their similarity to one another, and OTUs are defined based on the similarity threshold (usually 97% similarity) set by the researcher. Software: Usearch (vsesion 7.0, <http://drive5.com/uparse/>) Using QIIME (<http://qiime.org>).

1. In order to reduce the amount of redundant calculation in the process of analysis, non-repetitive sequences are need obtained from optimized dat (<http://drive5.com/usearch/manual/dereplication.html>）
2. Discarding singletons. (<http://drive5.com/usearch/manual/singletons.html>)
3. With 97% clustering, an OTU sequence should be at least 3% different from all other OTUs, and OTU representative sequence should be the most abundant sequences in its neighborhood. During the clustering process, chimeric sequences should be discarded.All the optimized sequence maps to OTU representative sequences are selected, and the sequences with 97% similarity to the representative sequences are selected to generate OTU tables.
4. Mapping all optimized sequences to OTU representation sequences, and sequences with a similarity＞97% are selected to generate an OTU table.
   1. Taxonomic analysis (domain, kingdom, phylum, class, order, family, genus, species)

The taxonomy of each 16S rRNA gene sequence was analyzed by Performances of Ribosomal Database Project (RDP) Classifier algorithm (vision 2.2) (<http://sourceforge.net/projects/rdp-classifier/>) against the SILVA 16S rRNA database (Release128 <http://www.arb-silva.de> using a confidence threshold of 70%).

- 1. Alpha diversity analysis

In ecology, alpha diversity (α-diversity) is the mean species diversity in sites or habitats at a local scale, it can reflect the richness and diversity of microbial communities.

Community richness index: sobs, chao, ace

Community diversity index: Shannon, simpson

Community coverage index: Coverage.

The indexes are described in detail as follows (<http://www.mothur.org/wiki/Calculators>). The alpha-diversities, including sobs, Shannon index (the Shannon diversity index), ACE estimator, Chao richness estimator, Simpson diversity index PD (Phylogenetic diversity) and Good’s coverage were calculated using Mothur (version: 1.30.1) ([http://www.mothur.org/wiki/Schloss_SOP#Alpha_diversity](http://www.mothur.org/wiki/Schloss_SOP#Alpha_diversity)).

Difference analysis of Alpha diversity --- For normally distributed data, one-way ANOVAs followed by LSD test were performed to compare between treatments. Non-normally distributed samples were subjected to the Kruskal Wallis test to compare between treatments. Normality was tested using the Shapiro-Wilk test. *P*-values≤0.05 were considered statistically signiﬁcant. These analyses were performed with the SAS 9.4 software package.

- 1. Rarefaction curve

In ecology, rarefaction is a technique to assess species richness from the results of sampling. Rarefaction allows the calculation of species richness for a given number of individual samples, based on the construction of so-called rarefaction curves. This curve is a plot of the number of species as a function of the number of samples. On the left, the steep slope indicates that a large fraction of the species diversity remains to be discovered. If the curve becomes flatter to the right, a reasonable number of individual samples have been taken: more intensive sampling is likely to yield only few additional species.

Software: mothur and R.

1. **Comparative Analysis**
   1. Beta diversity analysis
2. Hierarchical Clustering

Hierarchical clustering analysis is an algorithmic approach to find discrete groups with varying degrees of (dis)similarity in a data set represented by a (dis)similarity matrix (like Bray-Curtis distance matrix). These groups are clustered by hierarchical clustering algorithms based on the distance matrix, and presented as a dendrogram using the unweighted pair-group method with arithmetic averages (UPGMA). Software: Qiime (for calculating beta distance matrix), R.

1. Taxonomic heat maps

The difference of species’ abundance distribution in the samples can be quantified by the distance of statistics. Using the statistical algorithm to calculate the distance between each two samples and obtain the distance matrix, which can be used for further analysis of beta diversity and visual statistical analysis. Unweighted UniFrac algorithms can detect the presence of changes in samples, while weighted UniFrac algorithms can further quantify the variation of different lineages among samples.

Constructed using a complete linkage clustering method and Bray-Curtis distance metric. Performed using the vegan packages and *ggplot2* packages in R. Phylogenetic tree was reconstruct according Maximum Likelihood Estimate by using Fasttree (version 2.1.3 <http://www.microbesonline.org/fasttree/>）, FastUniFrac（<http://UniFrac.colorado.edu/）was> used to get the matrix distance between the samples.

1. PCoA analysis (Principal coordinates analysis),

is a constrained method of dimensionality reduction and it can explore as well as visualize similarities or dissimilarities of different samples’ community structure. PCoA is based on the chosen distance matrix. Both PCA and PCoA try to find the potential principal components that affect the composition of the sample community through dimensionality reduction. PCoA analysis, first sort a series of eigenvalues and eigenvectors, and then select the top important eigenvalues and show them in the coordinate system. The result is a rotation of the distance matrix, which does not change the relative position of the sample points, but only changes the coordinate system.

Software: R.

1. Non-metric multidimensional scaling analysis (NMDS)

NMDS analysis (Non-metric multidimensional scaling) is an indirect gradient analysis approach which produces an ordination based on a distance or dissimilarity matrix. Unlike methods which attempt to maximize the variance or correspondence between objects in an ordination, NMDS attempts to represent, as closely as possible, the pairwise dissimilarity between objects in a low-dimensional space. Any dissimilarity coefficient or distance measure may be used to build the distance matrix used as input. NMDS is a rank-based approach. This means that the original distance data is substituted with ranks. While information about the magnitude of distances is lost, rank-based methods are generally more robust to data which do not have an identifiable distribution.

Software: Qiime for calculating distance matrix, R vegan package for NMDS analyzing.

- 1. Enterotype analysis

Microbiota Typing Analysis mainly studies the type of dominant microflora in different samples through statistical clustering and ignores the influence of external factors such as environmental factors. This analysis can cluster different samples with similar dominant bacteria structure into one class, and it is mainly applicable to the microflora classification of specific environmental samples, such as enterotypes, cervicotypes and oral microbiota types.

Samples were clustered using Jensen-Shannon distance and partitioning around medoid (PAM) clustering based on the bacteria relative abundance. The optimal number of clusters was estimated using the Calinski-Harabasz (CH) index, Principal coordinates analysis (PCoA, K≥ 2) was carried out for visualization.

Software: R-- ade4 packages, cluster packages, clustersim packages.
